# Whole microbe arrays accurately predict interactions and overall antimicrobial activity of galectin-8 toward distinct strains of *Streptococcus pneumoniae*

**DOI:** 10.1038/s41598-023-27964-y

**Published:** 2023-04-01

**Authors:** Shang-Chuen Wu, Hau-Ming Jan, Mary L. Vallecillo-Zúniga, Matthew F. Rathgeber, Caleb S. Stowell, Kaleb L. Murdock, Kashyap R. Patel, Hirotomo Nakahara, Carter J. Stowell, Moon H. Nahm, Connie M. Arthur, Richard D. Cummings, Sean R. Stowell

**Affiliations:** 1 Joint Program in Transfusion Medicine, Department of Pathology, Brigham and Women’s Hospital, National Center for Functional Glycomics, 630E New Research Building, Harvard Medical School, 77 Avenue Louis Pasteur, Boston, MA 02115 USA; 2 Department of Medicine, University of Alabama at Birmingham, 1720 2nd Ave South Birmingham, Alabama, 35294 USA; 3 Harvard Glycomics Center, Beth Israel Deaconess Medical Center, Harvard Medical School, Boston, MA 02115 USA

**Keywords:** Microbiology techniques, Microbiome

## Abstract

Microbial glycan microarrays (MGMs) populated with purified microbial glycans have been used to define the specificity of host immune factors toward microbes in a high throughput manner. However, a limitation of such arrays is that glycan presentation may not fully recapitulate the natural presentation that exists on microbes. This raises the possibility that interactions observed on the array, while often helpful in predicting actual interactions with intact microbes, may not always accurately ascertain the overall affinity of a host immune factor for a given microbe. Using galectin-8 (Gal-8) as a probe, we compared the specificity and overall affinity observed using a MGM populated with glycans harvested from various strains of *Streptococcus pneumoniae* to an intact microbe microarray (MMA). Our results demonstrate that while similarities in binding specificity between the MGM and MMA are apparent, Gal-8 binding toward the MMA more accurately predicted interactions with strains of *S. pneumoniae,* including the overall specificity of Gal-8 antimicrobial activity. Taken together, these results not only demonstrate that Gal-8 possesses antimicrobial activity against distinct strains of *S. pneumoniae* that utilize molecular mimicry, but that microarray platforms populated with intact microbes present an advantageous strategy when exploring host interactions with microbes.

Cell surface carbohydrates are critical in cell–cell recognition, cell trafficking, cancer progression, and regulation of immune function^1–4^. In addition to representing a fundamental aspect of cellular regulation within a host, cell surface glycosylation can also play a critical role in host-microbial interactions^5–7^. As carbohydrates can completely envelope cell surfaces, microbial glycans are often the first molecular determinants recognized by host immunity^8^. Microbial glycans can be incredibly diverse, with the number of monosaccharides used as building blocks and the distinct forms of branching often far exceeding those used by mammals^9^. This diversity allowed serological reactivity alone to define individual strains of microbes long before genomic approaches were routinely used and likely aided in selective pressures that shaped adaptive immunity^10–12^.

While the overall diversity of microbial glycans far exceeds that observed on mammalian cells, some microbes instead decorate themselves with surface carbohydrates that mimic mammalian host cell glycans^13^. This approach may have resulted from evolutionary pressures that reduce the probability that a host can generate a robust humoral immune response secondary to immunological tolerance to self^13,14^. As adaptive immunity is therefore limited toward microbes that utilize molecular mimicry, innate immune factors that are hard-wired in the genome appear to fill this gap in adaptive immunity by specifically targeting microbes that utilize molecular mimicry. Recent studies suggest that the galectin family of glycan-binding proteins have a novel innate immune function that may have evolved in part to fill this gap in adaptive immunity by targeting microbes that utilize molecular mimicry^13,15^. In this way, galectins complement adaptive immunity by eliminating microbes that utilize molecular mimicry^13^.

Some of the most commonly recognized glycan determinants on the surface of mammalian cells are the ABO blood group antigens, which represent some of the earliest examples of host cell glycan structures mimicked by microbes. While immune tolerance to ABO(H) reduces the possibility of autoimmunity and allows the transfusion of blood products between compatible individuals^16,17^, failure to generate anti-A or anti-B antibodies creates a gap in adaptive immunity against microbes that likewise decorate themselves in blood group like antigens and illustrates the importance of innate immune factors that can fill this gap^13^. While prior studies have demonstrated that galectins can target microbes that utilize ABO(H) molecular mimicry, this form of molecular mimicry is not limited to ABO blood group antigens^14^. Among pathogens that utilize molecular mimicry, various strains of *Streptococcus pneumoniae* have also been described^18^. *S. pneumoniae* is particularly important as it is an infectious pathogen that causes respiratory infections in children and the elderly leading to millions of deaths worldwide^19^. In contrast to ABO(H) antigen-based molecular mimicry, *S. pneumoniae* microbial glycans can resemble structures uniformly expressed in all mammals and are therefore not polymorphic like ABO(H)^18^. As such, all individuals would be predicted to be tolerized to these forms of molecular mimicry, strongly suggesting that innate forms of immunity toward individual strains of *S. pneumoniae* may be particularly important.

Recent studies utilizing microbial glycan microarrays (MGMs), which are printed microarrays comprised of purified glycans from individual microbes, suggest that several innate immune galectins in particular may exhibit high specificity toward microbes that express self-like structures^20–27^. However, a possible limitation of the MGM approach, and perhaps glycan array utilization in general, is that the overall presentation of glycans following harvesting, purification and printing may not recapitulate the native glycan configuration that exists in situ on the cell surface. This raises the possibility that interactions observed following microarray analysis, while clearly useful in predicting interactions with intact cells, may not always accurately reflect the overall affinity of the given host immune factor for the target microbe they are designed to represent. To explore this possibility in detail, we leveraged previously employed strategies to fabricate microarrays^28–33^, including arrays populated with actual microbes that we have abbreviated as microbial microarrays (MMAs) to distinguish these arrays from the MGM. The goal of this study was to compare results obtained using the traditional MGM populated with glycans harvested from various strains of *S. pneumoniae* to the overall relative affinity and glycan binding specificity observed using MMAs. In these studies, we used galectin-8 (Gal-8), one of the most well-studied antimicrobial galectins, as a probe. Our results demonstrate that while similarities in the overall observed binding specificity between Gal-8 and each of its domains between the MGM and intact microbe microarray (MMA) were apparent, examination of Gal-8 binding toward the MMA most accurately predicted interactions with various strains of *S. pneumoniae,* including effective concentrations at which Gal-8 exerted its antimicrobial activity. Taken together, these results demonstrate that Gal-8 possesses antimicrobial activity against distinct strains of *S. pneumoniae* and that microarray platforms populated with intact microbes represents a useful approach when seeking to predict interactions with microbes in a high throughput manner.

## Results

### Galectin-8 exhibits specificity toward distinct strains of S. pneumoniae

Our previous studies demonstrate that several members of the galectin family display high affinity for a variety of microbial mimics of ABO(H) mammalian glycans^26,34–36^. These results suggested that galectins possess the ability to protect blood group positive individuals from blood group-positive microbes^15,34,36,37^. However, as molecular mimicry is not limited to blood group antigens, innate immunity would be predicted to extend beyond ABO(H) and related polymorphic antigens to engage a variety of microbial structures that resemble self. *S. pneumoniae* provides an example of this, where the distinct capsular polysaccharides of several strains of *S. pneumoniae* resemble invariant glycan motifs present on mammalian cells^19^. To define the binding specificity of galectins toward *S. pneumoniae* in more detail, while also exploring the capacity of current microarray strategies to accurately predict the binding specificity and overall antimicrobial of galectins, we defined the binding specificity of Gal-8 using a traditional MGM populated with 23 capsular polysaccharides (CPS) isolated from well-characterized strains of *S. pneumoniae* (Table S1)^18^.

**Figure 1:**
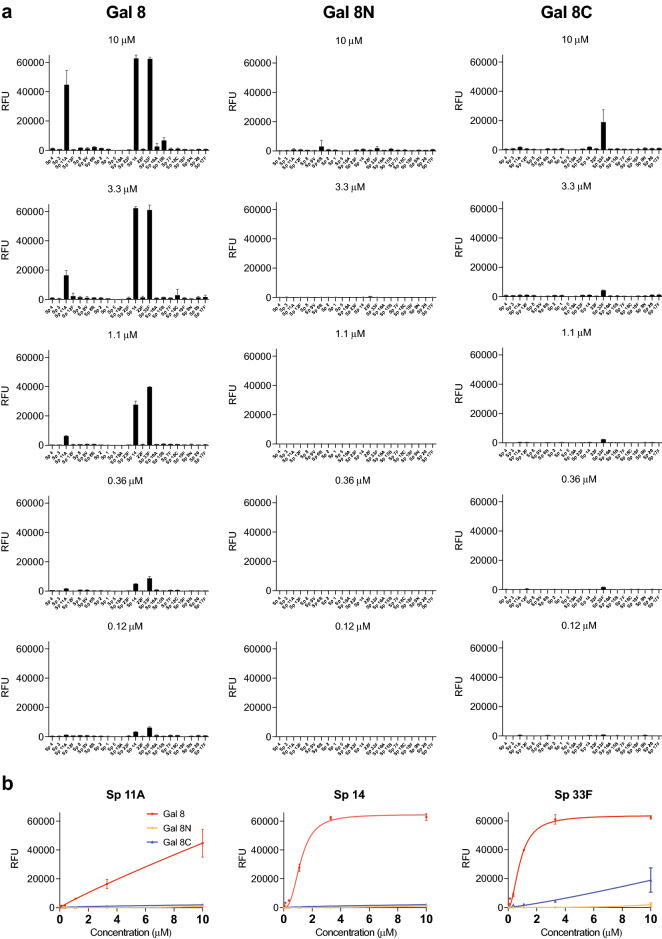
Gal-8 recognize *S. pneumoniae* that express self-like antigens on the microbial glycan microarray (MGM). (**a**) MGM array was incubated with Gal-8, Gal-8N and Gal-8C at the concentrations indicated. Error bars represent means ± standard deviation (SD). RFU, relative fluorescence units. *S. pneumoniae* Danish type 11A; Sp 11A, *S. pneumoniae* Danish type 14; Sp 14, *S. pneumoniae* Danish type 33F; Sp 33F. (**b**) Selected microbial glycans represented on the MGM were shown with binding isotherms. Error bars represent means ± SD.

Using this approach, we examined Gal-8 as well as its two independent domains, the N-terminal domain (Gal-8 N) and the C-terminal domain (Gal-8C) at a relatively low concentration (0.12 μM) in an effort to identify higher affinity microbial glycan interactions in this array format (Fig. 1a). Gal-8 exhibited binding toward microbial glycans isolated from *S. pneumoniae* Danish type 14 (Sp 14) and *S. pneumoniae* Danish type 33F (Sp 33F), both of which express variant forms of mammalian-like antigens. We then sought to define the capacity and overall binding specificity of Gal-8 over a range of concentrations beginning at 1.1 μM. We observed that Gal-8 displayed recognition of the CPS from *Streptococcus pneumoniae* Danish type 11A (Sp 11A) in addition to Sp 14 and Sp 33F. Importantly, the differential binding toward each of these strains was less apparent following analysis at 10 μM, a common single concentration used in prior studies to assess galectin-glycan interaction using microarray formats. Analysis of the individual domains of Gal-8 demonstrated that Gal-8C exhibited overlapping glycan-binding preferences with Gal-8 for Sp 33F, but with much weaker overall affinity. However, in contrast to Gal-8, no appreciable binding by Gal-8C toward the printed CPS of 14 or 11A could be detected. Unlike Gal-8C, Gal-8N failed to display detectable binding toward any of the microbial glycans represented on the array over the concentrations tested (Fig. 1a).

To ascertain the relative affinity of Gal-8 binding toward each of these microbial glycans, we analyzed binding isotherms generated following analysis of each concentration tested as recently outlined^34,37,38^. Using this approach, the observed *K*_D_ of Gal-8 toward Sp 14 and Sp 33F was very similar, around 1.2 ± 0.05 μM and 0.85 ± 0.05 μM, respectively (Fig. 1b). While binding toward 11A could be observed, the binding isotherm established over this concentration failed to reach saturation, preventing an accurate *K*_D_ estimate. While Gal-8C likewise exhibited increased binding toward Sp 33F with increased concentrations, this binding also never reached saturation and therefore was also not sufficient to allow *K*_D_ determination (Fig. 1b). Gal-8C and Gal-8N failed to exhibit sufficient binding toward any of the other Sp CPS glycans tested to generate a binding isotherm using this approach. These results strongly suggest that while the overall trends in bindings specificity were similar for Gal-8 and Gal-8C, Gal-8 exhibited higher affinity for the CPS of several *S. pneumoniae* strains compared with Gal-8C. Given the relative similarities in glycan recognition at the highest concentrations tested, these results also suggest that establishing binding isotherms may provide more accurate information regarding the relatively affinity of Gal-8 toward individual strains of *S.* pneumoniae^39^.

### MGM binding preferences do not fully align intact S. pneumoniae analysis

**Figure 2:**
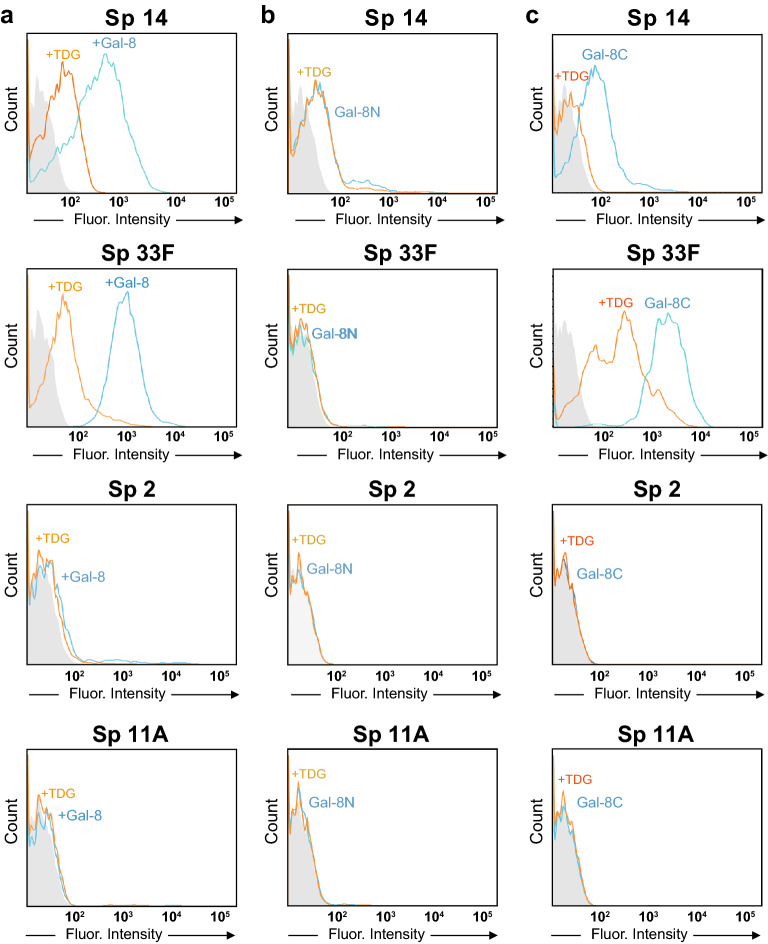
Gal-8 recognizes specific serotypes of *S. pneumoniae* on the flow cytometry analysis. (**a**, **b**, and **c**) Flow cytometric analysis of Gal-8 (**a**), Gal-8N (**b**), and Gal-8C (**c**) binding to Sp 14, 33F, 2 and 11A with or without inclusion of final concentration 50 mM TDG as indicated. TDG is labeled in orange. Unstained control is labeled in gray. Each respective galectin is labeled in blue.

The typical purpose of glycan microarray analysis is to predict interactions of a protein, e.g., lectin or antibody, with specific cell surface glycans. Recent studies indicate that MGM array results may accurately predict interactions of host innate immune proteins, including galectins, toward intact microbes^34,36^. However, as this approach has typically been confined to MGM analysis at a single concentration^26^, we next sought to define whether binding isotherm analysis on the MGM may more accurately predict actual interactions with intact microbes^34,36,37^. To accomplish this, we examined Gal-8 binding toward intact strains of *S. pneumoniae* by flow cytometric analysis (Fig. 2). Similar to MGM observations, Gal-8 readily bound Sp 14 and Sp 33F and this binding was inhibited by thiodigalactoside (TDG), a non-metabolizable inhibitor of galectin-glycan interactions (Fig. 2a)^40^, demonstrating that these interactions require glycan recognition. As a control we examined possible Gal-8 interactions with *S. pneumoniae* Danish type 2 (Sp 2), a strain of *S. pneumoniae* that Gal-8 failed to recognize following CPS isolation and printing in the MGM format. Similar to results from MGM studies, no detectable binding toward Sp 2 was observed by flow cytometry, strongly suggesting that the MGM results predict the specificity of Gal-8 toward various strains of *S. pneumoniae*.

However, while Gal-8 readily engaged Sp 11A in the MGM format, virtually no detectable binding could be observed toward Sp 11A by flow cytometry (Fig. 2a). However, this difference could reflect examination of Gal-8 binding by flow cytometric analysis at 0.1 μM, a concentration where very little binding was likewise observed toward Sp 11A in the MGM format. Similar to MGM analysis, Gal-8N failed to recognize Sp 14, 33F, 2 or 11A (Fig. 2b), while Gal-8C displayed some binding toward Sp 33F similar to the MGM. However, unlike MGM findings, Gal-8C bound to Sp 14 (Fig. 2c). Taken together, these results suggest that while the Gal-8 analysis on the MGM can provide general information about glycan binding specificity toward distinct strains of *S. pneumoniae*, it may not fully predict actual interactions with intact microbes.

### MGM binding preferences do not fully align Gal-8 anti-microbial activity

**Figure 3:**
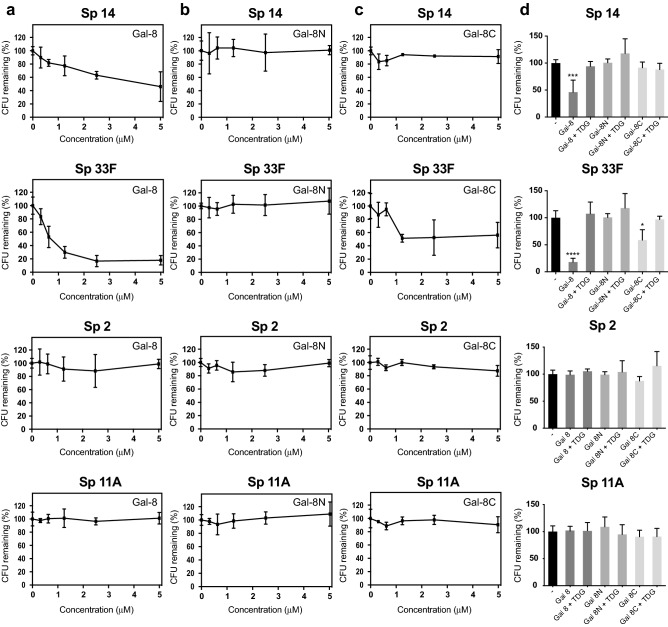
Measuring the impact of Gal-8 on the microbial viability (**a**, **b**, and **c**) Colony forming units (CFUs) remaining after incubation with the indicated concentrations of Gal-8 (**a**), Gal-8N (**b**), and Gal-8C (**c**). (**d**) Quantification of viable bacteria after incubation with PBS control, 5 μM Gal-8 or each of its domains with or without 50 mM TDG as indicated. Data are represented as mean values ± SD. Statistical analysis was performed using one-way ANOVA with Tukey’s test. *P* values less than 0.05, 0.001, and 0.0001 are summarized with one, three, and four asterisks, respectively.

Given the differences observed in Gal-8 binding between MGM analysis and flow cytometric examination of intact microbes, we next sought to define which approach might more accurately predict antimicrobial activity. To test this, we first evaluated the antimicrobial activity of Gal-8 toward Sp 14 and Sp 33F. Incubation with Gal-8 resulted in significant loss of viability of both strains (Fig. 3a). However, the highest level of Gal-8 antimicrobial activity and overall potency exhibited significant strain dependency; Sp 33F was much more sensitive to the antimicrobial activity of Gal-8 when compared to Sp 14, despite similar overall binding characteristics on the MGM. Consistent with the inability of Gal-8 to bind Sp 2 following both MGM analysis and flow cytometric detection, Gal-8 likewise failed to impact the viability of Sp 2 when evaluated in parallel. However, in contrast to MGM findings, Gal-8 not only failed to engage Sp 11A (Fig. 2a), but likewise did not exhibit any antimicrobial activity over the concentrations tested (Fig. 3a). Similar to MGM results and flow cytometric analysis, Gal-8N failed to impact the viability of Sp 33F, 14, 2 or 11A (Fig. 3b). Gal-8C exhibited some killing activity toward Sp 33F (Fig. 3c), similar to binding observed on the MGM (Fig. 1). However, Gal-8C failed to significantly alter the viability of Sp 14, 11A or 2 (Fig. 3c), consistent with predicted outcomes observed on the MGM (Fig. 1), but not entirely consistent with the ability of Gal-8C to possess at least some binding toward Sp 14 following flow cytometric examination (Fig. 2c). In each setting where antimicrobial activity was observed, inclusion of TDG inhibited Gal-8 antimicrobial activity, demonstrating that antimicrobial activity depended on Gal-8-glycan interactions (Fig. 3d). These results demonstrate that while the MGM and flow cytometric analysis can be used to detect Gal-8 interactions with microbial glycans, the capacity of the MGM or flow cytometry to fully predict antimicrobial activity toward individual strains of *Streptococcus pneumoniae* may be limited.

### The MMA more accurately predicts Gal-8 binding and antimicrobial activity

In the above studies we observed that flow cytometric approaches may provide a useful strategy to define possible interactions with distinct microbial strains. Consistent with this, except for the outcomes of Gal-8C binding toward Sp 14 (Fig. 2c), flow cytometric examination appeared to be more accurate in predicting antimicrobial activity toward distinct strains of *Streptococcus pneumoniae* than the MGM. Flow cytometric examination, however, requires analysis of individual microbes in an isolated fashion and therefore does not lend itself to the same high throughput analysis provided by glycan microarrays. Flow cytometric analysis is also further limited by challenges interpreting data over a range of concentrations when seeking to establish binding isotherms that may provide a relative affinity measurement toward a given microbe. However, glycan presentation following isolation and printing in an array format may differ from the configuration observed on the cell surface, possibly accounting in part for distinct glycan binding outcomes observed when compared to antimicrobial activity; such challenges are not entirely unique to the MGM and may reflect factors that could influence array analysis outcomes in any glycan microarray format.

**Figure 4:**
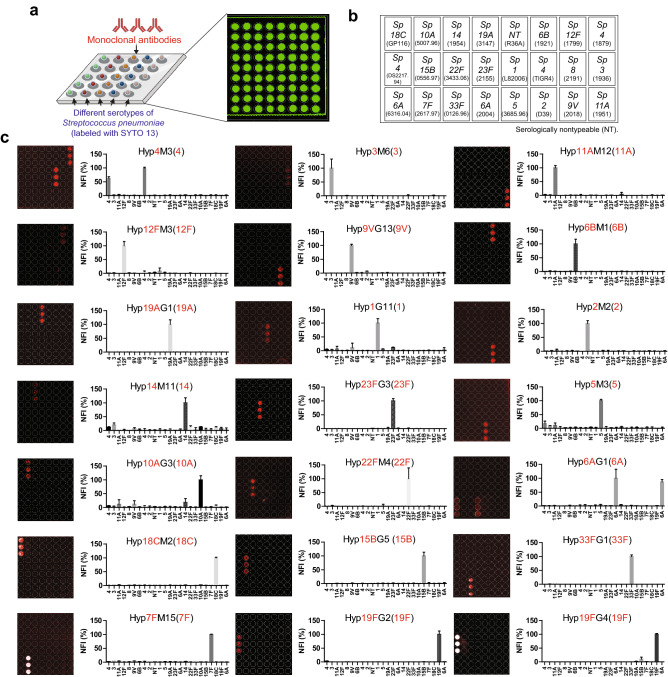
Validation of MMA using defined monoclonal antibodies. (**a**) Schematic overview for the MMA. Bacteria labeled with SYTO 13 were printed to the nitrocellulose glass slide, and serotype specific monoclonal antibodies were then added to the microarray following Alexa Fluor 647 conjugated secondary antibody detection. (**b**) Printing layout of MMA. Isolated *S. pneumoniae* without a capsule was defined as serologically nontypeable (NT).^89^ Serotypes were shown and strain name is labeled in the parentheses. (**c**) Left panel of each figure indicate images of fluorescent signals observed on the microarray. Their corresponding targeted serotype of each antibody was labeled in red. All fluorescent intensities were normalized by the highest intensity value and shown as Normalized fluorescent intensity (NFI). Data shown correspond to the mean of triplicate spots and error bars represent means ± SD.

To address limitations in MGM-based analysis, we next sought to leverage the strengths of the microarray platform with the ability to interrogate glycan interactions in situ on the microbe surface. To accomplish this, we utilized a similar strategy as employed previously to examine galectin-glycan interactions toward other cells and microbes in general^28–33,41–43^. This was done by growing each individual microbe, followed by fixation and labeling with the DNA binding dye, SYTO 13, to provide a qualitative approach to determine whether each microbe could be detected following microarray fabrication (Fig. 4a)^28–33^. Following growth, fixation and labeling, each strain of microbe was printed in a microarray format (Fig. 4b). To assess the utility of this overall approach, we first sought to define whether the distinct carbohydrate antigens that distinguish each individual strain *S. pneumoniae* remained accessible following fixation, STYO 13 staining and printing in this format. To this end, we probed the array with a series of defined monoclonal antibodies directed against distinct glycan determinants present on the surface of individual strains of *S. pneumoniae* to simply determine whether the predicted microbe could be detected in the array format using this approach*.* Each antibody recognized the predicted strain of *S. pneumoniae* in the MMA format*,* with the only cross-reactivity occurring between distinct strains known to share common glycan motifs recognized by the respective monoclonal antibody (Fig. 4c). These results demonstrate that individual strains of *S. pneumoniae* can be fixed, labeling and printed in a microarray format and that each strain retains the ability to be engaged by monoclonal antibodies directed against specific epitopes on their glycan coat.

**Figure 5:**
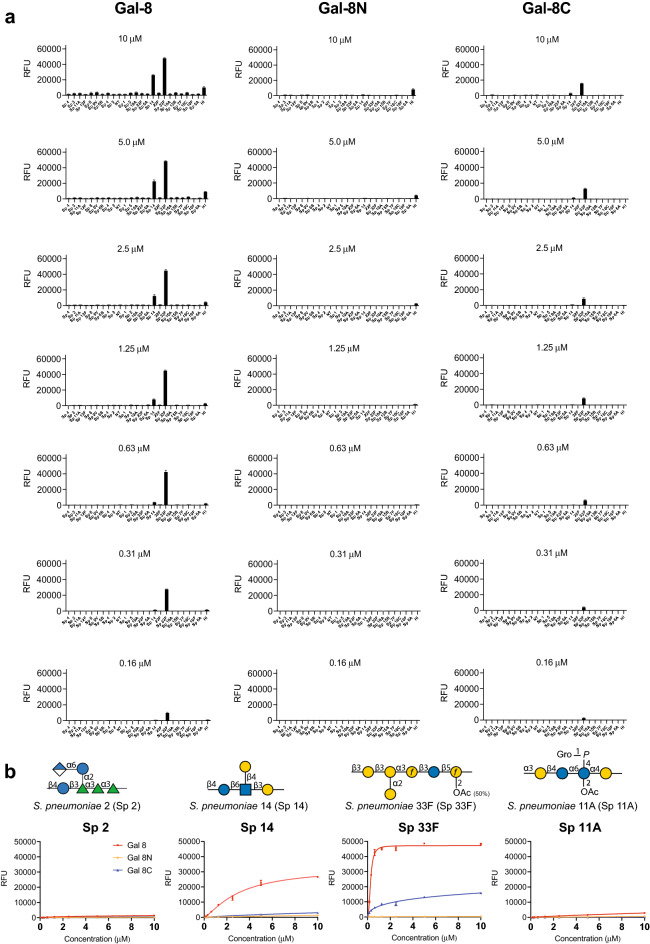
Gal-8 recognize specific strain of *S. pneumoniae* that express self-like antigens on the MMA. (**a**) MMA was incubated with Gal-8, Gal-8 N and Gal-8C at the concentrations indicated. HI: * Haemophilus influenzae.* Error bars represent means ± standard deviation (SD). RFU, relative fluorescence units. (**b**) Selected serotypes of *S. pneumoniae* represented on the MMA were shown with binding isotherms. Microbial glycan structures for each respective microbe are shown. Error bars represent means ± SD.

As the purpose in generating the MMA was to develop a high throughput approach that may more accurately assess the overall activity of Gal-8 toward individual strains of *S. pneumonia,* we next sought to determine whether Gal-8 could recognize *S. pneumoniae* in this array format and if so, whether the observed relative affinity matched the overall binding characteristics observed by the MGM, flow cytometric analysis and/or its antimicrobial activity. To accomplish this, we first examined Gal-8 at the relatively low concentration (Fig. 5a), analogous to our initial examination of Gal-8 binding toward the MGM. Similar to the MGM, binding was observed by Gal-8 toward microbes printed in the MMA. However, in contrast to the MGM, Gal-8 binding was particularly apparent initially toward Sp 33F, followed by Sp 14 (Fig. 5a, S1 and S2b). Unlike the outcome observed on the MGM, no appreciable binding was observed toward Sp 11A, similar to the outcome observed following flow cytometric analysis. Similar to Gal-8N analysis on the MGM, Gal-8N likewise failed to display appreciable binding on the *S. pneumonia* MMA (Fig. 5b), but did recognize *Haemophilus influenzae* (HI), which served as positive control for Gal-8 and Gal-8N^31^. In contrast to results obtained following flow cytometric examination of Gal-8C toward Sp 14, Gal-8C failed to recognize Sp 14 in the MMA format, although it did bind to Sp 33F, largely in agreement with MGM findings. Inclusion of TDG preventing Gal-8 and Gal-8C MMA interactions, demonstrating that the binding observed in this format was dependent on protein-glycan interactions (Figure S2c).

To further explore the possible ability of the MMA to predict the antimicrobial activity of Gal-8 toward individual strains of *S. pneumoniae,* we next evaluated the corresponding binding isotherms generated following evaluation of Gal-8, Gal-8 N and Gal-8C (Fig. 5b). In contrast to the relatively similar affinities observed by Gal-8 toward the isolated and printed CPS structures of Sp 33F and 14 (*K*_Ds_ of 0.85 ± 0.05 μM and 1.2 ± 0.05 μM, respectively), Gal-8 exhibited a much higher affinity toward Sp 33F when evaluated in the MMA format than the MGM format, where the *K*_Ds_ of 0.27 ± 0.01 μM and 3.07 ± 0.43 μM toward Sp 33F and Sp 14, respectively, were observed. While binding of Gal-8C toward Sp 33F was observed, the binding was not sufficient to reliably establish a *K*_D_. No appreciable binding between Gal-8N and the MMA could be observed. Taken together, these results suggest that microarray platforms populated with intact microbes may provide a useful strategy when seeking to accurately predict actual interactions with target microbes in a high throughput manner and that Gal-8 exhibits antimicrobial activity toward distinct strains of *S. pneumoniae* that utilize molecular mimicry.

## Discussion

Hosts interact with microbes through engagement of a variety of molecular determinants^44^. While carbohydrates reside at the surface of microbes as the first determinants often engaged by host immune factors, the complexity of these structures has also made the study of host interactions with microbial glycans challenging to study^45^. This has resulted in limited data regarding the overall specificity of host immune factors, particularly innate immune lectins, toward microbial glycans. This is especially apparent when considering the diversity of glycans within a given species as evident in the present study with *S. pneumoniae*^46^*.* The importance of these cell surface glycans in the pathogenesis of *S. pneumoniae* is evident by the role of the capsule in overall *S. pneumoniae* virulence as well as the efficacy of vaccine approaches designed to protect individuals from *S. pneumoniae* based on eliciting immune responses against these distinct capsular glycan structures^46,47^. Thus, the glycan coat plays a critical role in the virulence of *S. pneumoniae* and the ability of host to combat *S. pneumoniae* infection. Fortunately, many elegant studies have developed a variety of microarray platforms to define the binding specificity of innate and adaptive immune factors for microbial glycans^28–33^. Through the use of these and similar platforms, host interactions with microbial glycans and the overall consequences of these interactions are beginning to be defined^25,28–33,48^.

While adaptive immunity can be tailored to protect a host from *S. pneumoniae* through vaccination or prior infection^49,50^, structures on microbes that resemble self may represent a challenge to host immunity in general due to reduced targeting ability against motifs that resemble host-like structures. As adaptive immunity is predicted to be limited in this scenario, innate immune factors, which are not subject to the same tolerance programs, may have evolved to protect individuals from microbes that utilize molecular mimicry to evade humoral immunity^13^. Prior examples of innate immunity against molecular mimicry primarily focused on ABO(H) blood group antigens^15,26,36,51^. This was in part due to differences in anti-A and anti-B antibody production based on polymorphic ABO(H) blood group status that had been recognized for over a century^52^. While RBC polymorphisms are certainly not limited to ABO(H) antigens and antibodies can certainly be generated against other alloantigens^53–59^, the development of antibodies against these structures are unique in that they spontaneously form in the first few months of life^17,60,61^. Although a variety of hypotheses have existed regarding the role of microbes in the genesis of anti-ABO(H) antibodies^62^, early studies suggested that several microbes exist that can decorate themselves in blood group-like antigens^52,62^. While these prior studies provided a possible source of antigen exposure in the development of anti-ABO(H) antibodies, they also raised important questions regarding how individuals who are ABO(H) blood group positive and therefore do not generate anti-ABO(H) antibodies protect themselves from these microbes. Prior results suggest that galectins can recognize and kill blood group positive microbes^15,34,36,37,63^, providing one example of possible protection against molecular mimicry. These data, coupled with the present results, strongly suggest that this form of innate immunity is not limited to blood group positive microbes, but possibly extends to other forms of molecular mimicry. In doing so, the present findings, coupled with prior data, begin to establish galectins more broadly, and Gal-8 in particular, as key players in host immunity against molecular mimicry.

The ability of galectins to bind very specific strains of microbes sets them apart from almost all other innate immune factors. In contrast to galectins, most host innate immune factors evolved the ability to engage microbes through recognition of common motifs found on many different microbes^64,65^. This property likely evolved to avoid the possible impact of diversification of antigenic structures on the surface in preventing microbes from being detected by host immune cells. Indeed, the ability to recognize common microbial motifs is highly conserved throughout evolution^65–67^. Engagement of such motifs by a series of innate immune receptors often serves as a key signaling event, alerting sentinel immune cell of microbial invasion, and directly linking innate and adaptive immunity^68,69^. In this setting, recognition of a microbial motif that is clearly distinct from self is critical to appropriately activate host defenses against an invading pathogen and is therefore a classic example of hard-wired self-non-self discrimination^65–69^. In contrast, the selectivity of galectins toward distinct strains of microbes resembles antibody-like specificity and sets them apart from most other hard-wired host immune proteins. Galectins likewise contrast both innate and adaptive immunity as they appear to exclusively engage microbe structures that resemble self. This specificity is likely required for the ability of galectins to fill this important gap in adaptive immunity and sets them apart from other forms of host immune function.

Despite the distinct nature of galectin-mediated host immunity, the mechanism whereby galectins exert their antimicrobial activity remains to be fully defined. Prior studies have demonstrated the ability of galectins, in particular, Gal-8, to compromise the membrane integrity of blood group positive *E. coli.*^15^ Differences in the membrane composition of prokaryotes and eukaryotes may in part account for the distinct sensitivity of bacteria versus mammalian cells to galectin-killing activity through distinct interactions that may occur with the phospholipid membrane that results in loss of membrane integrity and ultimately death. Such an ability has been described for antimicrobial peptides in general and may reflect a general feature galectin antimicrobial activity^70–72^. However, it is possible that the mechanism of galectin killing occurs completely independent of direct interactions with the microbial membrane; future studies will certainly be needed to define mechanisms whereby galectins exert their antimicrobial activity.

While the results of the present study illustrate the unusual ability of galectins to target individual strains of microbes, analysis of the binding specificity of innate immune lectins or even antibodies against the wide range of individual strains of a given microbe in parallel can often prove challenging. Unlike many other innate immune factors that often engage microbial motifs commonly shared between many microbial species^65–69^, examination of the glycan binding specificity toward many strains often requires the analysis of many distinct microbes in parallel^7^. The development of glycan microarrays has been transformative in the evaluation of lectin-glycan interactions in general, owing to the synthetic complexity of glycan biosynthesis and the ability of microarray platforms to be generated with relatively little glycan material when compared to other biochemical approaches^28–33,73^. However, despite the nearly uniform application of this approach, questions have certainly remained regarding whether results obtained using this format accurately reflect actual interactions with the intact microbes. Differences in printing efficiency, in addition to possible differences in glycan presentation, may in part account for potential differences between array results and actual interactions with the same glycan motifs presented on a cell surface^15,28–34,36,37^. However, while limitations still exist in these platforms, glycan microarrays, including MMA-like strategies^28–33^, appear to possess the ability to serve as reasonable screening tools when seeking to predict general features of host immune factor recognition of intact microbes or cells.

The present results demonstrate the MGM format is useful and has the capacity to convey important information regarding the overall binding specificity of Gal-8. Of the glycans analyzed on this array format, Gal-8 exhibited a high level of specificity for distinct glycan determinants, which appears to largely mirror observations following examination of Gal-8 toward intact microbes. However, differences in the binding specificity and overall affinity were apparent following a direct comparison between MGM results and analysis of intact microbes represented on MMA. While this variability may appear to be relatively subtle, when seeking to establish the network of host interactions with distinct strains of microbes, the relative affinity and overall consequences of galectins toward individual strains of microbes is important as such results would be predicted to have implications when interpreting the possible outcome of Gal-8 interactions toward individual strains of microbes. While flow-based approaches appeared to more accurately predict actual interactions with microbes, results obtained following analysis of the Gal-8C domain alone also demonstrated binding by flow cytometry that was not apparent on the MMA and did not translate to antimicrobial activity. While this outcome could easily reflect insufficient binding to trigger death of the target microbes, it is intriguing that analysis of whole microbe printing in the MMA more accurately predicted the lack of antimicrobial activity exhibited by Gal-8C toward Sp 14. Similarly, while similar affinity was observed by Gal-8 toward Sp 33F and Sp 14 on the MGM, very different results were obtained following analysis of the same intact microbes in the MMA format by Gal-8.

The distinct glycan binding preferences observed provide insight into intrinsic aspects of Gal-8 glycan recognition. However, interpretation of the impact of discrete glycan modifications on the surface of microbes can be difficult to fully interpret given the diversity of structures present and the unique variations of glycan modifications on microbial surfaces. For example, type 2 LacNAc presented on the capsular polysaccharides on the Sp 14 may support Gal-8 recognition as this LacNAc is likewise a common constituent on the mammalian cell surface^74^. However, the presentation of the type 2 LacNAc glycan on Sp 14 is distinct from what occurs on mammalian cells and this difference may result in distinct binding affinities. Similarly, while Gal-8 recognized the CPS of Sp 33 and the intact Sp 33 microbe, the CPS of Sp 33 is unique from most mammalian glycans. The Galβ1-3Gal linkage may ultimately be responsible for supporting glycan binding, but it is not clear to what extent the Galβ1-2 linkage may likewise influence Gal-8 binding to this microbe^75^. Importantly, Gal-8 also bound *Haemophilus influenza,* consistent with recent findings^26,31^. As noted, the synthetic complexity of microbial glycans prohibits a detailed analysis of the unique glycan determinants that are ultimately responsible for dictating differences in binding affinity observed by Gal-8 toward unique microbial glycans. This can be further complicated by challenges associated with accurately defining glycan or microbial printing efficiency that can make it difficult to interpret relative binding preferences when results are obtained at a single concentration^38^. By examining galectin binding over a range of concentrations a relative affinity can be obtained that may provide additional information regarding the overall preference of a given galectin for a distinct microbial strain^38^. However, array results ultimately require confirmation with intact microbes. Importantly, MMA findings most accurately predicted actual antimicrobial activity. These results suggest that glycan presentation in situ is an important determinant of the fine specificity of Gal-8 toward individual strains of *S. pneumoniae* and that these differences are relevant when considering the antimicrobial activity of this protein.

In summary, our results demonstrate that Gal-8 possess remarkable specificity toward individual strains of *S. pneumoniae* and that its ability to bind and kill target strains appears to reflect an example of innate immunity against molecular mimicry. Equally important, these findings also suggest that microarray platforms populated with intact microbes as in the MMA may provide a complementary strategy to traditional glycan microarray technology when seeking to identify the binding specificity of a host innate immune protein.

## Materials and methods

### Protein expression and purification

Expression plasmids encoding human Gal-8, Gal-8N, and Gal-8C were transformed into *E.coli* BL21 (DE3), and then expressed as described previously^15,34,76–78^. Briefly, bacteria transformed with plasmid were grown in LB broth containing 100 μg/mL ampicillin with 250 rpm at 37 °C. When bacteria were grown to the mid-log phase, protein expression was induced by two distinct conditions. For Gal-8 and Gal-8N, bacteria were added by 1 mM isopropyl 1-thio-β-D-galactopyranoside (IPTG) and followed by 16 °C for 20 h incubation. For Gal-8C, 0.4 mM IPTG and 22 °C incubation for 20 h were used. After protein induction, 6 L cultured bacteria were pelleted and then resuspended in 60 mL bacterial lysis buffer (PBS with 14 mM 2-mercaptoethanol (2-ME), 60 μL ribonuclease A (RNase A), 60 μL DNase I, 60 μL lysozyme, and 2 protease inhibitor cocktail tablets). The bacteria were lysed using sonication-cooling cycles of 10 s on and 10 s off for total 1 h. Bacterial cell debris were removed by centrifugation at 17,000 rpm at 4 °C for 1 h twice. Supernatant was then applied to lactosyl-sepharose column. Target protein fractions were eluted by PBS with 14 mM 2-ME and 100 mM lactose. When performing biological assays, 2-ME and lactose were removed using a PD-10 gel filtration column (Cytiva).

### Bacterial strains

*Streptococcus pneumoniae* strains were kindly provided by Dr. Moon H. Nahm (Department of Medicine, University of Alabama at Birmingham, Birmingham, Alabama, USA). *S. pneumoniae* strains were cultivated on trypticase soy agar II blood agar plates (TSA II -BA; Becton Dickson BBL) or on Todd-Hewitt yeast broth (Todd-Hewitt broth with 0.5% yeast extract) at 37 °C in an atmosphere of 5% CO_2_ using AnaeroPouch-MicroAero bag (Mitsubishi Gas Chemical Company, Inc). Colonies were transferred to Todd-Hewitt yeast broth and incubated at 37 °C and 5% CO_2_ until the culture reached exponential growth phase (OD600 of ∼0.4), followed by isolation and preparation for MMA incorporation. *Haemophilus influenzae* (HI) GAP 516 was a clinically isolated strain and was cultured on chocolate agar plate (Remel, R01301) at 37 °C in an atmosphere of 5% CO_2_ using AnaeroPouch-MicroAero bag.

### Microbial glycan microarrays (MGM array)

MGM (version 5.2) glycan microarray was obtained from the National Center for Functional Glycomics (NCFG), funded by the National Institute of General Medical Sciences (NIGMS) of the National Institutes of Health (NIH). MGM array was prepared as described previously^26,73^. 2 mg/mL Gal-8, Gal-8N or Gal-8C was labeled with 1 mg Alexa Fluor 647 N-Hydroxysuccinimide (NHS) Ester with the addition of lactose (100 mM final concentration) to help maintain the stability and incubated for 1 h at room temperature and avoid from light as outlined previously^79^. Free lactose and unconjugated Alexa Fluor 647 were removed by a PD-10 gel filtration column. To remove possible inactive protein, labeled Gal-8, Gal-8N or Gal-8C were purified again by lactosyl-sepharose column. Bound Gal-8, Gal-8N or Gal-8C were eluted with 100 mM lactose in PBS plus 2-ME. Notably, lactose was then removed using PD-10 gel filtration column and stored at 4 °C in PBS with 14 mM 2-ME prior to the use of Gal-8 for microarray experiments. Ultimately, labeled Gal-8, Gal-8N or Gal-8C were applied to printed MGM microarray as described previously^34,36,37,80,81^. Slides were incubated with fluorescent labeled Gal-8, Gal-8N or Gal-8C at the concentrations indicated in Tris buffer/Salts/Metal ions (TSM) Binding Buffer (20 mM Tris–HCl, 150 mM sodium chloride, 2 mM calcium chloride, 2 mM magnesium chloride, pH 7.4, 1% BSA, and 0.05% Tween 20, with 14 mM 2-ME) for 1 h at room temperature in a dark humid chamber. Slides were similarly incubated with strain specific monoclonal antibodies. Slides were washed by successive immersion in TSM containing 0.05% Tween 20 (4x), TSM (4x), and water (4x)^82^. All slides were dried by microarray slide spinner, and an image of bound fluorescence was obtained using a microarray scanner (GenePix 4000 B, Molecular devices). Integrated spot intensities were acquired using Imagene software (GenePix Pro 7)^83,84^. The complete dynamic range of the GenePix 4000 B scanner is between the range of intensities (RFU) on the image from 1 to > 65,000. Array analyses were performed in a linear range so that saturation was not reached in each experiment, allowing analysis of the binding isotherm of binding without signal saturation.

### *S. pneumoniae* intact microbe microarray (MMA)

Bacterial cells were fixed with 1% paraformaldehyde for 24 h at 4 °C and then washed with PBS. Bacterial cells were labeled by 5 μM SYTO™ 13 (S7575, Invitrogen) for 15 min at room temperature in the dark. After labeling, bacterial cells were washed with PBS, resuspended to OD600 of ~ 1 and printed onto Nitrocellulose Film-Slides (Grace bio-labs, 705,108). Proteins were directly labeled with Alexa Fluor 647 (Thermo, A20006) and diluted into different concentration by PBST (1 × PBS, 0.05% Tween-20) with 1% BSA. Slides were blocked with SuperG blocking buffer (Bio-labs) for 1 h at room temperature. Slides were likewise incubated with varying concentrations of each galectin as indicated for 1 h in a dark chamber at room temperature. For defined monoclonal antibodies, which were provided by Dr. Moon H. Nahm, slides were incubated with serotype specific antibodies, followed by detection anti-mouse IgG conjugated with Alexa Fluor 647 (1:100) (Jackson, 115-605-164). Fluorescence images were acquired using a microarray scanner (GenePix 4000 B, Molecular devices). Integrated spot intensities were acquired using Imagene software (GenePix Pro 7).

### Flow cytometry analysis

Bacteria were cultured to mid-logarithmic phase and adjust to OD600 of 0.1. Bacterial cells were then washed twice with PBS at 4 °C and then incubated with 0.1 μM Alexa Fluor 647 labeled proteins with or without 50 mM TDG at 4 °C for 10 min. Bacteria were washed with PBS twice and resuspended in 400 μl PBS for flow cytometry analysis using FACS Canto II flow cytometer (BD Biosciences)^85–87^. The data were processed by FlowJo version 10.

### Measuring the impact of Gal-8 on the bacterial viability

Bacteria were grown to mid-logarithmic phase and then diluted into OD600 of 0.1. Bacterial cells were suspended with the indicated concentrations of Gal-8 or each of its domain at 37 °C for 2 h. The presence of Colony forming unit (CFU) was detected by limited dilution analysis^88^.

## Supplementary Information

Supplementary Information.

## Data Availability

All data are reported in the main text or in the supplemental information of this work. Any additional information required to reanalyze the data reported in this paper is available from the corresponding author upon request.
