# Supplementary material for: Whole microbe arrays accurately predict interactions and overall antimicrobial activity of galectin-8 toward distinct strains of Streptococcus pneumoniae
Source: Sci Rep. 2023 Apr 1;13:5324. doi: 10.1038/s41598-023-27964-y (PMC10067959; doi:10.1038/s41598-023-27964-y)
Supplement: Supplementary file 1 — Supplementary Information. [file 41598_2023_27964_MOESM1_ESM.pdf]

Figure S1

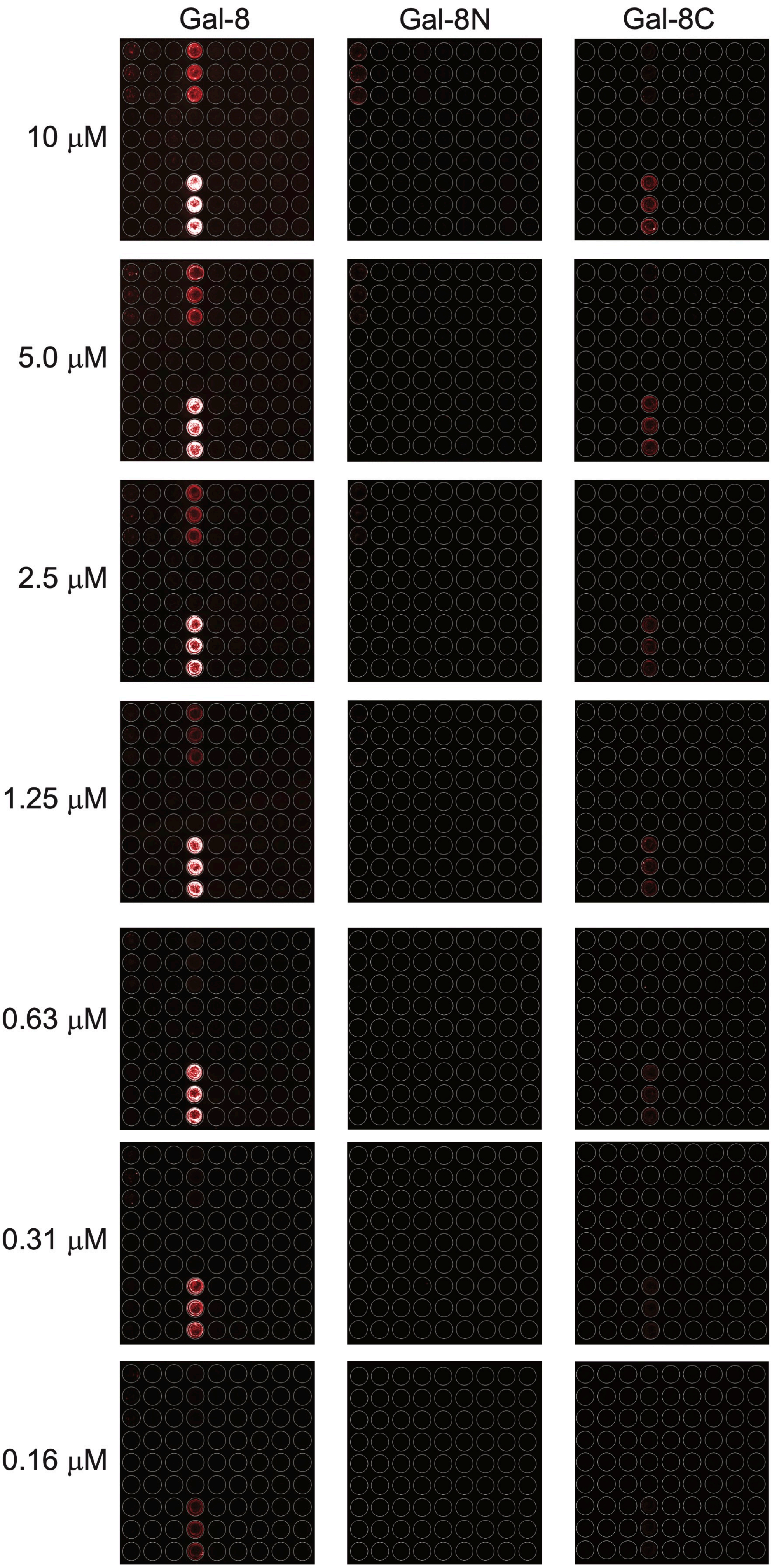

Figure S2

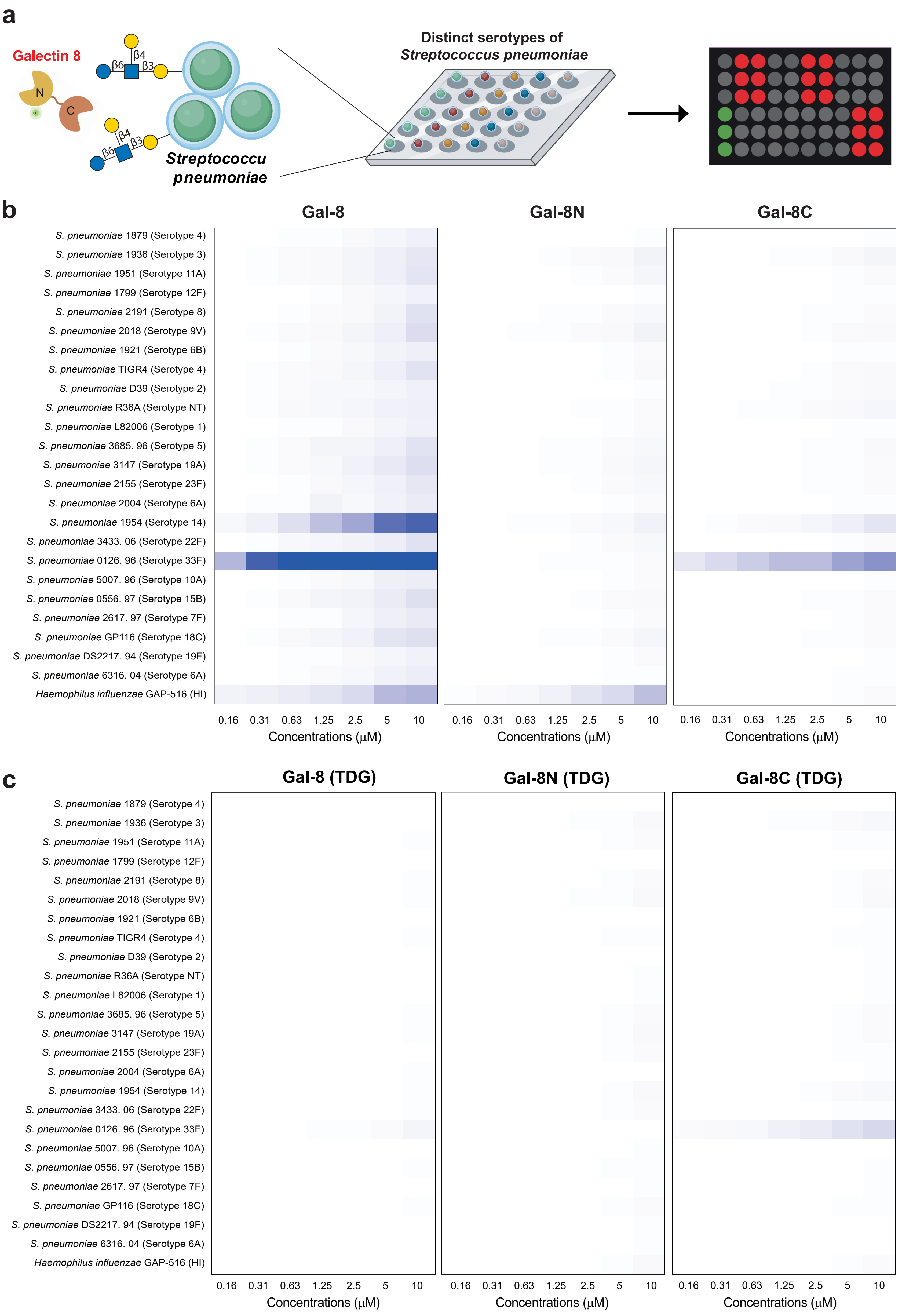
a
Galectin 8
N
C
β4
β6
β3
Streptococcu
pneumoniae
Distinct serotypes of
Streptococcus pneumoniae
b
Gal-8
Gal-8N
Gal-8C
S. pneumoniae 1879 (Serotype 4)
S. pneumoniae 1936 (Serotype 3)
S. pneumoniae 1951 (Serotype 11A)
S. pneumoniae 1799 (Serotype 12F)
S. pneumoniae 2191 (Serotype 8)
S. pneumoniae 2018 (Serotype 9V)
S. pneumoniae 1921 (Serotype 6B)
S. pneumoniae TIGR4 (Serotype 4)
S. pneumoniae D39 (Serotype 2)
S. pneumoniae R36A (Serotype NT)
S. pneumoniae L82006 (Serotype 1)
S. pneumoniae 3685. 96 (Serotype 5)
S. pneumoniae 3147 (Serotype 19A)
S. pneumoniae 2155 (Serotype 23F)
S. pneumoniae 2004 (Serotype 6A)
S. pneumoniae 1954 (Serotype 14)
S. pneumoniae 3433. 06 (Serotype 22F)
S. pneumoniae 0126. 96 (Serotype 33F)
S. pneumoniae 5007. 96 (Serotype 10A)
S. pneumoniae 0556. 97 (Serotype 15B)
S. pneumoniae 2617. 97 (Serotype 7F)
S. pneumoniae GP116 (Serotype 18C)
S. pneumoniae DS2217. 94 (Serotype 19F)
S. pneumoniae 6316. 04 (Serotype 6A)
Haemophilus influenzae GAP-516 (HI)
0.16 0.31 0.63 1.25 2.5 5 10
Concentrations (μM)
c
Gal-8 (TDG)
Gal-8N (TDG)
Gal-8C (TDG)
0.16 0.31 0.63 1.25 2.5 5 10
Concentrations (μM)

Table S1 Schematic representation of Streptococcus pneumoniae carbohydrate determinants. Type represent Danish type.

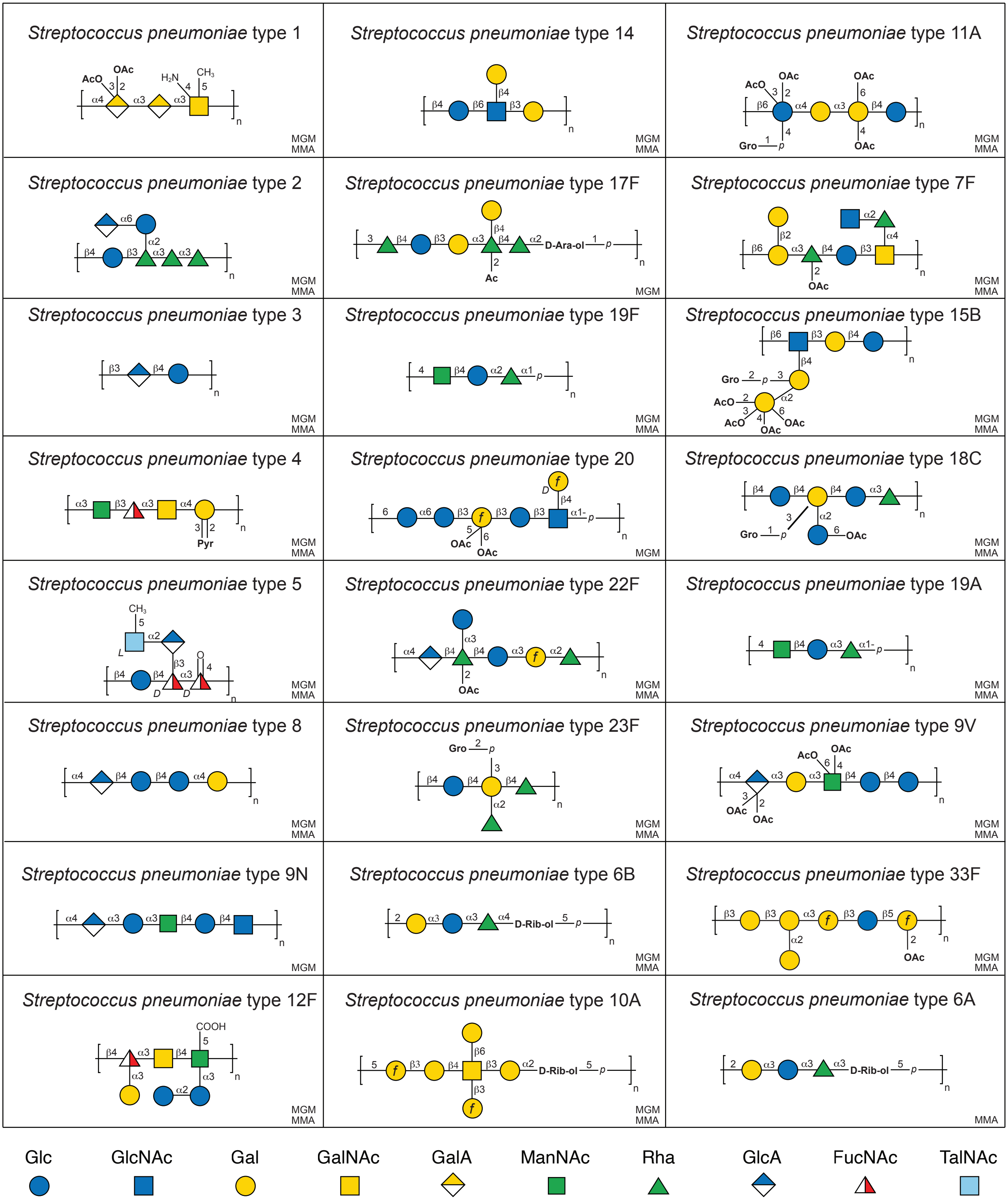

Figure S1. Binding of Gal-8, Gal-8N, and Gal-8C to the MMA. Bacteria were printed as triplicates and the binding was detected using Alexa Fluor 647-labeled galectins. Representative microarray images of Gal-8, Gal-8N and Gal-8C over a range of concentrations starting from 0.16 to 10 μM were used on the MMA.

Figure S2. Gal-8 recognizes specific strains of *S. pneumoniae* that express self-like antigen on the MMA. (**a**) A schematic overview of MMA. Bacteria were grown to an exponential phase followed by 1% paraformaldehyde fixation. After labeling with SYTO 13, bacteria were printed onto a nitrocellulose glass slide, following interrogation with Alexa Fluor 647 labeled Gal-8. Fluorescent signal was obtained by the microarray scanner and shown as RFU (relative fluorescence units). (**b and c**) Heatmaps for the RFU values of Gal-8, Gal-8N and Gal-8C with (b) or without (c) TDG over a broad range of concentrations starting from 0.16 to 10 μM on the MMA. The higher RFU values shown are dark blue, while the lowest RFU values shown are white.
